# Supplementary material for: Tomato Bushy Stunt Virus Nanoparticles as a Platform for Drug Delivery to Shh-Dependent Medulloblastoma
Source: Int J Mol Sci. 2021 Sep 29;22(19):10523. doi: 10.3390/ijms221910523 (PMC8509062; doi:10.3390/ijms221910523)
Supplement: Supplementary file 1 [file ijms-22-10523-s001.zip › Lico et al Table S1.pdf]

Table S1. List of the peptides fused to the TBSV CP

**Peptide Name**	**Peptide Sequence**
RGD	*GGPGG*-RGD
RiGiD	*GGPGG*-ARGDK-**GPG**-ARGDK-
CooP	*GGPGG*-CGLSGLGVA
tLyp1	*GGPGG*-CGNKRTR
ApoE	*GGPGG*- LRKLRKRLLLRKLRKRLL

The linker sequence is highlighted in italic, the spacer sequence is in bold.
