# Supplementary material for: Tomato Bushy Stunt Virus Nanoparticles as a Platform for Drug Delivery to Shh-Dependent Medulloblastoma
Source: Int J Mol Sci. 2021 Sep 29;22(19):10523. doi: 10.3390/ijms221910523 (PMC8509062; doi:10.3390/ijms221910523)
Supplement: Supplementary file 1 [file ijms-22-10523-s001.zip › Lico et al Table S4_REVISED.pdf]

Table S4 – MM/PBSA Results.

**Receptor**	**Ligand**	**Binding Free Energy (ΔG, kJ/mol)**			
		**0 – 25 ns**	**25 – 50 ns**	**50 – 75 ns**	**75 – 100 ns**
NRP-1	tLyp1	-109.64 +/-101.47	-104.56 +/- 93.00	-86.60 +/- 71.98	-43.23 +/- 78.39
	CP-tLyp1	-3.80 +/- 83.15	-74.11 +/- 85.42	-86.97 +/- 91.77	-91.07 +/- 74.47
FABP3	CooP	-118.27 +/- 25.75	-118.20 +/-22.96	-128.41 +/-18.41	-129.30 +/- 21.66
	CP-CooP	-502.9 +/- 68.07	-457.36+/108.67	-391.34 +/-101.20	-377.07 +/- 131.58

Note: Binding Free Energy (ΔG) of the binding sites of receptors in complex with the free peptides and the peptides fused to the CP of TBSV, as predicted by MM/PBSA calculations. The values clearly show that CooP peptide fused to the viral CP recognize with almost 3-fold higher binding affinity the FABP3 receptor (ΔG = -377.07 kJ/mol vs ΔG = -129.30 kJ/mol).
