# Supplementary material for: Tomato Bushy Stunt Virus Nanoparticles as a Platform for Drug Delivery to Shh-Dependent Medulloblastoma
Source: Int J Mol Sci. 2021 Sep 29;22(19):10523. doi: 10.3390/ijms221910523 (PMC8509062; doi:10.3390/ijms221910523)
Supplement: Supplementary file 1 [file ijms-22-10523-s001.zip › Lico et al Table S5_REVISED.pdf]

Table S5. List of the oligonucleotides used for peptides cloning, molecular screening and PCR/qPCR analysis.

Name	5' --> 3' Sequence
5'tLyp1	CGGAGGTTGTGGAAATAAGAGAACTAGGTGAGAGCTCTTAAT
3'tLyp1	TAAGAGCTCTCACCTAGTTCTCTTATTTCCACAACCTCCGGGCC
5'CooP	CGGAGGTTGTGGTCTTTCTGGATTGGGAGTTGCTTGAGAGCTCTTAAT
3'CooP	TAAGAGCTCTCAAGCAACTCCCAATCCAGAAAGACCACAACCTCCGGGCC
5'RiGiD	CGGAGGTGGGCCCGGAGGAGGTGCTAGAGGAGATAAGGGTCCTGGTGCTAGGGGTGACAAATGAGAGCTCTTAAT
3'RiGiD	TAAGAGCTCTCATTTGTCACCCCTAGCACCAGGACCCTTATCTCCTCTAGCACCTCCTCCGGGCCCACCTCCGGGCC
5'ApoE	CGGAGGTCTTAGAAAGTTGAGGAAAAGATTGCTTCTCAGGAAGTTAAGGAAACGACTTCTGTGAGAGCTCTTAAT
3'ApoE	TAAGAGCTCTCACAGAAGTCGTTTCCTTAACTTCCTGAGAAGCAATCTTTTCCTCAACTTTCTAAG ACCTCCGGGCC
5'RGD	CGGAGGTAGAGGAGATTGATTAAT
3'RGD	TAATCAATCTCCTCTACCTCCGGGCC
TBSV-For	TGACATTTCGTGCAACTGG
TBSV-Rev	AAGATCCAAGGACTCTGTGC
qTBSV-For	CGGTTGCAGTCACCAGACAA
qTBSV-Rev	CCCAGACGTCCTGCCAGTA
qGADPH-For	CATGGCCTTCCGTGTTCCTA
qGADPH-Rev	GCGGCACGTCAGATCCA
qβ-ACTIN-For	CACAGCTTCTTTGCAGCTCCTT
qβ-ACTIN-Rev	CGTCATCCATGGCGAACTG
qRlp13a-For	CTGCTCTCAAGGTTGTTCGGCT
qRlp13a-Rev	CCTTCCGTTTCTCCTCCAGAGT
qRlp32-For	TGTGCAACAAATCTTACTGTGCT
qRlp32-Rev	TGCACACAAGCCATCTACTCA
qTBP-For	GAAGAACAATCCAGACTAGCAGCA
qTBP-Rev	CCTTATAGGGGAACTTCACATCACAG
