# Supplementary material for: Tomato Bushy Stunt Virus Nanoparticles as a Platform for Drug Delivery to Shh-Dependent Medulloblastoma
Source: Int J Mol Sci. 2021 Sep 29;22(19):10523. doi: 10.3390/ijms221910523 (PMC8509062; doi:10.3390/ijms221910523)
Supplement: Supplementary file 1 [file ijms-22-10523-s001.zip › Lico et al Suppl M&M_REVISED.pdf]

SUPPLEMENTARY MATERIAL

Tomato Bushy Stunt Virus nanoparticles as a platform for drug delivery to Shh-dependent medulloblastoma

Authors

Chiara Lico[1§], Barbara Tanno[2§], Luca Marchetti[2], Flavia Novelli[2], Paola Giardullo[2], Caterina Arcangeli[3], Simonetta Pazzaglia[2], Maurizio S. Podda[3], Luca Santi[4], Roberta Bernini[4], Selene Baschieri[1*] and Mariateresa Mancuso[2*]

Molecular Docking and Molecular Dynamics Simulations

The structural models of the asymmetric unit of the cTBSV in which at the C-terminal of each CP the tLyp1/CooP peptide was inserted by means of a GGPGG linker have been generated by homology modeling. The template consisted of the atomic coordinates of TBSV (PDB id: 2TBV (A, B, C), [1, 2]. The calcium ions, which are known to play an important role in the stability of the viral particle, were also included during the construction of the models. The cTBSV models were then generated by expanding the asymmetric unit using the 60 transformation matrices provided by VIPERdb database tools [3; http://viperdb.scripps.edu]. All structural models used or homology builded or obtained by docking procedure and used as starting structure for the MD simulations are available at GitHub link: https://github.com/mspodda/TBSV-nanoparticles-as-a-platform-for-drug-delivery-to-Shh-dependent-medulloblastoma-PDB-models.

The MD simulations were performed by using the all-atom AMBER 99sb-ildn force field [4] for the peptides and proteins. The force field parameters for oleic acid (OLA) ligand were generated using the LigPar server [5-7]. Most of the systems (tLyp1-NRP-1, tuftsin-NRP-1, CPtLyp1-NRP-1 and CPCooP-FABP3) were placed in a dodecahedric box, whereas the remaining systems (CooP-FABP3 and OLA-FABP3) were placed in a cubic box and filled with TIP3P water model [8]. All the systems were neutralized using NaCl at the physiological concentration of 150 mM. Periodic boundary conditions were applied to avoid edge effects. The systems were energy minimized by steepest descent (SD) [9], heated and equilibrated for 400 ps. MD simulations were carried out in the NPT ensemble for 100 ns, each step of 2 fs. The v-rescale algorithm [10] was used to keep the temperature at 310 K (tt=0.1 ps). The average pressure was kept at 1 bar (tp=2 ps) by using the isotropic Parrinello-Rahman barostat [11]. The particle mesh Ewald (PME) method was used to compute the long-range electrostatics and Van der Waals interactions with a cut-off of 1 nm. An equal cut-off was used for short-range electrostatics and Van der Waals interactions. Rotation and translational motions of systems were removed, and all bonds were constrained with the LINCS algorithm [12]. Initial velocities were assigned according to Maxwell-Boltzmann distribution.

Cluster analysis was performed with the standard gromos-clustering algorithm [13]. The method counts the number of neighbours for each structure using a root mean square displacement (rmsd) cutoff distance (<0.15 nm) and assigns the structure with the largest number of neighbours along with all its neighbours to a cluster. Once assigned to a cluster, the structures are discharged from the pool of structures. The procedure is repeated until the exhaustion of all the structures. Hydrogen bond (H-bond) analyses were performed using VMD. An H-

bond was assumed to exist during the simulation if the donor to acceptor distance was shorter than 0.35 nm and the hydrogen-donor-acceptor distance was shorter than 30°. Absolute binding free energies were evaluated through the MM/PBSA (molecular mechanics/Poisson-Boltzmann solvent accessible surface area) method by using the g mmpbsa tool of GROMACS [14, 15]. The average free energies of solvation (ΔGbinding) between the residues of the active site of the receptors and the tLyp1 and CooP peptides (both in their free version or as fusion to the viral CP) were calculated for four different temporal windows: 0-25 ns, 25-50 ns 50 – 75 ns and 75-100 ns.

References

1. Harrison S.C., Olson A.J., Schutt C.E., Winkler F.K., Bricogne G. Tomato bushy stunt virus at 2.9 A resolution. *Nature* **1978**, *276*, 368-373.
2. Olson A.J., Bricogne G., Harrison S.C. Structure of tomato bushy stunt virus IV. The virus particle at 2.9 A resolution. *J Mol Biol* **1983**, 171:61-93.
3. Carrillo-Tripp M., Shepherd C.M., Borelli I.A., Venkataraman S., Lander G., Natarajan P., Johnson J.E., Brooks C.L. 3rd, Reddy V.S. VIPERdb2: an enhanced and web API enabled relational database for structural virology. *Nucleic Acids Res* **2009**, *37*, D436-442.
4. Lindorff-Larsen K., Piana S., Palmo K., Maragakis P., Klepeis J.L., Dror R.O., Shaw D.E. Improved side-chain torsion potentials for the Amber ff99SB protein force field. *Proteins* **2010,** *78*:1950-8.
5. Jorgensen W.L., Tirado-Rives J. Potential energy functions for atomic-level simulations of water and organic and biomolecular systems. *Proc Natl Acad Sci USA* **2005**, *102*, 6665-6670.
6. Dodda L.S., Cabeza de Vaca I., Tirado-Rives J., Jorgensen W.L. LigParGen web server: an automatic OPLS-AA parameter generator for organic ligands. *Nucleic Acids Res* **2017** *45*, W331-W336.
7. Dodda L.S., Vilseck J.Z., Tirado-Rives J., Jorgensen W.L. 1.14*CM1A-LBCC: Localized Bond-Charge Corrected CM1A Charges for Condensed-Phase Simulations. *J Phys Chem B* **2017**, *121*, 3864-3870.
8. Jorgensen W.L., Chandrasekhar J., Madura J.D., Impey R.W., Klein M.L. Comparison of simple potential functions for simulating liquid water. *J Chem Phys* **1983**, *79*, 926-935.
9. Petrova S.S., Solov'ev A.D. The Origin of the Method of Steepest Descent. *Hist Math* **1997**, *24*, 361-375.
10. Bussi G., Donadio D., Parrinello M. Canonical sampling through velocity rescaling. *J Chem Phys* **2007**, *126*, 014101.
11. Parrinello M., Rahman A. Polymorphic transitions in single crystals: A new molecular dynamics method. *J Appl Phys* **1981**, *52*, 7182-7190.
12. Hess B, Bekker H, Berendsen HJC, Fraaije JGEM. LINCS: A linear constraint solver for molecular simulations. *J Comp Chem* **1997**, *18*, 1463-1472.
13. Daura X., Gademann K., Jaun B., Seebach D., van Gunsteren W.F., Mark A.E. Peptide Folding: When Simulation Meets Experiment. *Angewandte Chemie International Edition* **1999**, *38*, 236-240.
14. Baker N.A., Sept D., Joseph S., Holst M.J., McCammon J.A. Electrostatics of nanosystems: Application to microtubules and the ribosome. *Proc Natl Acad Sci USA* **2001**, *98*, 10037-10041.
15. Kumari R., Kumar R., Open Source Drug Discovery Consortium, Lynn A. g_mmpbsa--a GROMACS tool for high-throughput MM-PBSA calculations. *J Chem Inf Model* **2014**, *54*, 1951-1962.
