# Supplementary material for: Tomato Bushy Stunt Virus Nanoparticles as a Platform for Drug Delivery to Shh-Dependent Medulloblastoma
Source: Int J Mol Sci. 2021 Sep 29;22(19):10523. doi: 10.3390/ijms221910523 (PMC8509062; doi:10.3390/ijms221910523)
Supplement: Supplementary file 1 [file ijms-22-10523-s001.zip › Lico et al Figure S2_REVISED.pptx]

## Slide 1
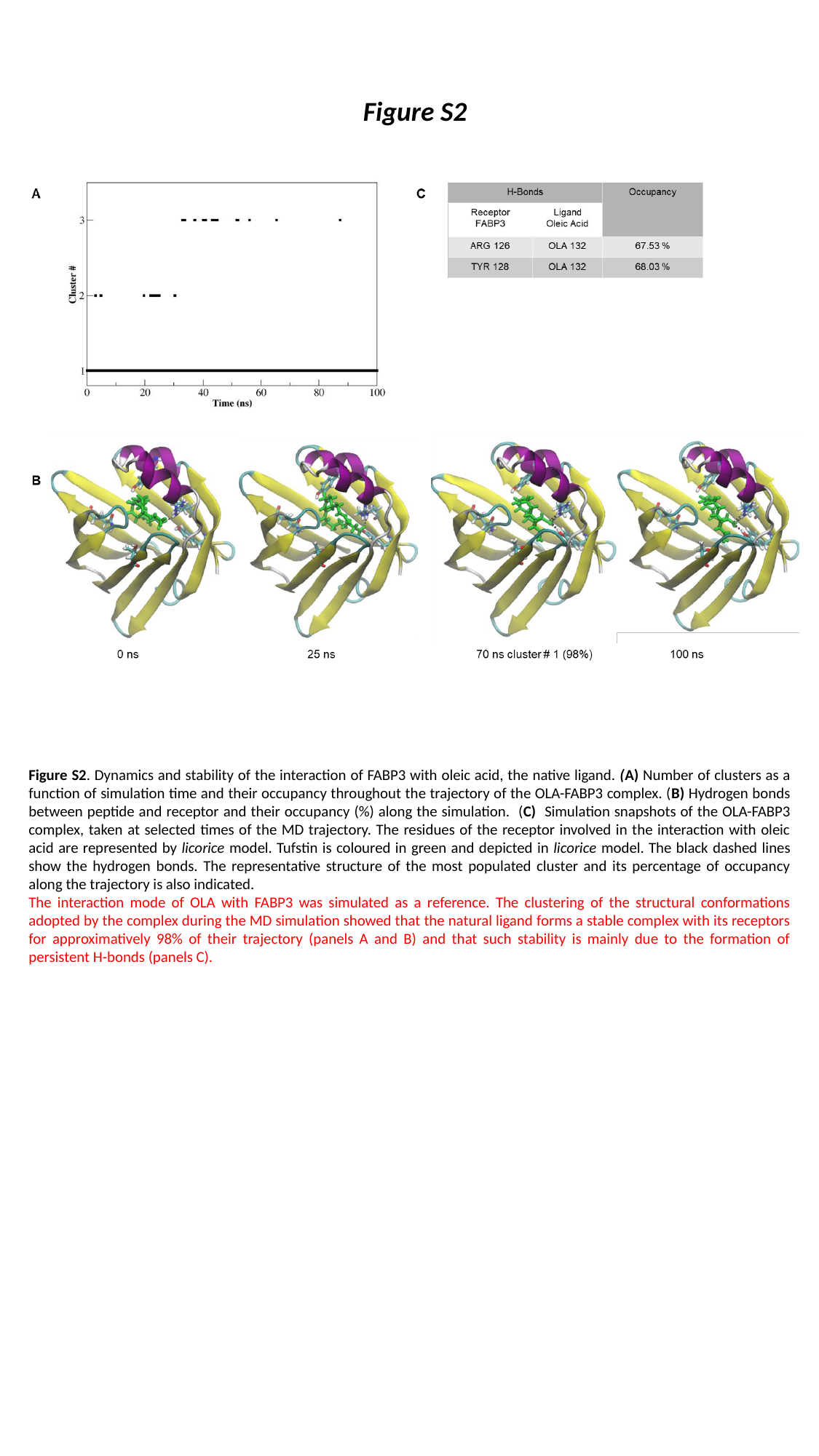

Figure S2
Figure S2. Dynamics and stability of the interaction of FABP3 with oleic acid, the native ligand. (A) Number of clusters as a function of simulation time and their occupancy throughout the trajectory of the OLA-FABP3 complex. (B) Hydrogen bonds between peptide and receptor and their occupancy (%) along the simulation. (C) Simulation snapshots of the OLA-FABP3 complex, taken at selected times of the MD trajectory. The residues of the receptor involved in the interaction with oleic acid are represented by licorice model. Tufstin is coloured in green and depicted in licorice model. The black dashed lines show the hydrogen bonds. The representative structure of the most populated cluster and its percentage of occupancy along the trajectory is also indicated.
The interaction mode of OLA with FABP3 was simulated as a reference. The clustering of the structural conformations adopted by the complex during the MD simulation showed that the natural ligand forms a stable complex with its receptors for approximatively 98% of their trajectory (panels A and B) and that such stability is mainly due to the formation of persistent H-bonds (panels C).
