# Supplementary material for: Tomato Bushy Stunt Virus Nanoparticles as a Platform for Drug Delivery to Shh-Dependent Medulloblastoma
Source: Int J Mol Sci. 2021 Sep 29;22(19):10523. doi: 10.3390/ijms221910523 (PMC8509062; doi:10.3390/ijms221910523)
Supplement: Supplementary file 1 [file ijms-22-10523-s001.zip › Lico et al Table S2_REVISED.pdf]

Table S2 – Molecular Docking Results.

ClusPro[(a)] or HADDOCK[(b)] complexes	Receptor	Ligand	ΔG (kcal/mol)	H-Bonds	
				receptor	ligand
tLyp1 – NRP1[(a)]	NRP-1	tLyp1 peptide	-8.9	ASP 320	ARG 5
				ASP 320	THR 6
				TYR 353	LYS 4
CPtLyp1 – NRP1[(a)]	NRP-1	tLyp1 peptide fused to CP of TBSV	-8.7	TYR 297	LYS 4
				PRO 317	ARG 7
				ASP 320	ARG 5
				ASP 320	ARG 7
				GLU 319	GLY393 (L)
				TYR 322	ASN385
				ASN 323	LEU386
tufstin – NRP1[(a)]	NRP-1	tufstin peptide (natural ligand)	-7.0	TYR 297	LYS 2
				TYR 297	PRO 3
				ASP 320	ARG 4
				LYS 351	ARG 4
CooP – FABP3[(a)]	FABP3/ H-FABP	CooP peptide	-12.4	TYR 19	GLY 7
				ALA 75	LEU 6
				GLN 95	LEU 6
				ARG 126	ALA 9
CPCooP – FABP3[(a)]	FABP3/ H-FABP	CooP peptide fused to CP of TBSV	-12.7	ASN 59	PRO390(L)
				ALA 75	LEU 6
OLA – FABP3[(b)]	FABP3/ H-FABP	oleic acid (natural ligand)	-9.4	TYR 19	OLA 133
				TYR 128	OLA 133

Note: HADDOCK tool was used to obtain the OLA-FABP3 complex, since ClusPro is not suitable for docking chemicals, such as oleic acid (OLA), to proteins. Binding free energies (ΔG) and hydrogen bonds (H-Bonds) between receptors and ligands of the best ClusPro or HADDOCK derived docked complexes as calculated by Prodigy webserver.
