# Supplementary material for: Tomato Bushy Stunt Virus Nanoparticles as a Platform for Drug Delivery to Shh-Dependent Medulloblastoma
Source: Int J Mol Sci. 2021 Sep 29;22(19):10523. doi: 10.3390/ijms221910523 (PMC8509062; doi:10.3390/ijms221910523)
Supplement: Supplementary file 1 [file ijms-22-10523-s001.zip › Lico et al Table S3_REVISED.pdf]

Table S3 – H-Bonds Analysis.

**tLyp1**			**tLyp1 fused to CP of TBSV**		
**H-Bonds**		**Occupancy**	**H-Bonds**		**Occupancy**
NRP-1	***tLyp1***		***NRP-1***	***tLyp1***	
GLN 296	ARG 7	19.25 %	PRO 317	ARG 7	70.94 %
TYR 297	LYS 4	14.43 %	GLY 318	ARG 5	60.16 %
TYR 297	ARG 7	14.54 %	GLU 319	ARG 5	59.22 %
GLY 318	ARG 7	41.70 %	GLU 319	ASN 3	51.88 %
GLU 319	ARG 7	16.92 %	ASP 320	ARG 7	93.93 %
ASP 320	ASN 3	28.57 %	LYS 351	ARG 7	59.55 %
ASP 320	ARG 5	43.96 %	***Residues of CP of TBSV***		
ARG 323	ARG 7	11.56 %	GLY 375	ASP 191	30.98 %
THR 413	ARG 5	13.74 %	LYS 397	ASP 191	37.65 %

**CooP**			**CooP fused to CP of TBSV**		
**H-Bonds**		**Occupancy**	**H-Bonds**		**Occupancy**
FABP3	***CooP***		***FABP3***	***CooP***	
TYR 19	GLY 7	90.39 %	THR 53	LEU 6	46.85 %
THR 60	GLY 2	10.49 %	ARG 106	GLY 7	67.38 %
GLN 95	GLY 7	38.99 %	ARG 126	LEU 3	57.78 %
ARG 126	ALA 9	100.00 %	TYR 128	SER 4	49.94 %
			Residues of CP of TBSV		
			LYS 52	ASP 341	46.58 %

Note: H-Bonds between peptides and receptors and their occupancy (%) along the simulation. The H-Bonds with occupancy > 40% along the trajectory are underlined. The binding to NRP-1 and of FABP3 by their peptides fused to the TBSV CP seems to be stronger compared to the free peptides as assessed by the higher H-Bonds occupancy values.
